# A Three-Domain Scoring System to Customize the Risk of Relapse of Differentiated Thyroid Carcinoma

**DOI:** 10.3390/cancers13174335

**Published:** 2021-08-27

**Authors:** Arnoldo Piccardo, Giacomo Siri, Martina Ugolini, Francesco Fiz, Matteo Puntoni, Gianluca Bottoni, Ugo Catrambone, Fabián Pitoia, Pierpaolo Trimboli

**Affiliations:** 1 Department of Nuclear Medicine, E.O. “Ospedali Galliera”, 16128 Genoa, Italy; arnoldo.piccardo@galliera.it (A.P.); martina.ugolini@galliera.it (M.U.); gianluca.bottoni@galliera.it (G.B.); 2 Scientific Directorate, Galliera Hospital, 16128 Genoa, Italy; giacomo.siri@galliera.it; 3 Clinical & Epidemiological Research Unit, University Hospital of Parma, 43126 Parma, Italy; matteo.puntoni@galliera.it; 4 Department of Surgery, E.O. “Ospedali Galliera”, 16128 Genoa, Italy; ugo.catrambone@galliera.it; 5 Division of Endocrinology, Hospital de Clínicas, University of Buenos Aires, Buenos Aires C1053, Argentina; fpitoia@intramed.net; 6 Servizio di Endocrinologia e Diabetologia, Ospedale Regionale di Lugano, Ente Ospedaliero Cantonale (EOC), 6900 Lugano, Switzerland; 7 Facultà di Scienze Biomediche, Università della Svizzera Italiana (USI), 6900 Lugano, Switzerland

**Keywords:** domains, scoring system, DTC, RAI, pre-RAI Tg, post-therapeutic ^131^-I-WBS

## Abstract

**Simple Summary:**

In this study, we have identified and validated a large series of patients affected by DTC, and created a three-domain scoring system able to identify the risk of persistence-relapse of disease after initial treatment (e.g., thyroidectomy and RAI). This three-domain system includes potential prognostic factors such as demographic (age and gender) and RAI-related (pre-RAI Tg levels and the post-therapeutic ^131^I WBS) data. This score is easy to calculate and interpret, as it provides a score ranging from 0 to 100; it allows clinicians to identify those patients who need stricter clinical surveillance or proper treatment due to a risk of disease persistence/relapse. This prognostic system can be used to semi-quantify the recurrence risk.

**Abstract:**

Purpose: the validation of a new scoring model considering the principal risk factors of differentiated thyroid cancer (DTC) relapse. Methods: we evaluated all DTC patients treated with thyroidectomy and radioactive iodine (RAI) therapy. Three domains were considered: the demographic domain (age and gender), the surgical domain (histology and the American Thyroid Association risk categories), and the RAI-related domain (pre-RAI thyroglobulin and post-therapeutic ^131^I whole-body scan). The progression-free survival was assessed. The patients’ sample was randomly split into a training and validation set. The three-domain score was calculated as the weighted sum of the levels of each significant factor, then scaled to an integer range (0–100) and, finally, stratified into terciles: mild risk 0–33, moderate risk 34–66, and severe risk 67–100. Results: 907 DTC patients were included. The RAI-related domain was the most relevant factor in the score calculation. The tercile stratification identified significantly different survival curves: patients within the two upper terciles showed approximately 6 to 30 times more progressive risk than patients at mild risk. Conclusion: we have validated a three-domain scoring system and the principal impact on this score is provided by the peri-RAI findings, whose prognostic role seems to be essential in risk identification.

## 1. Introduction

Differentiated thyroid carcinoma (DTC) is the most common endocrine malignancy, whose incidence and prevalence have been steadily increasing over the last decades [1,2]. DTCs are considered to be among the least lethal of all cancer forms [3,4,5]; however, disease relapse is a relatively frequent occurrence, which might affect up to 20% of DTC patients [6] and negatively impact the overall quality of life of these subjects [7]. For these reasons, it is pivotal to obtain an accurate assessment of the DTC aggressiveness at the time of diagnosis and directly follow this with the necessary treatment. The 2015 American Thyroid Association (ATA) guidelines proposed a three-tiered (high, intermediate, and low-risk) stratification [6]. This system considers the TNM staging, the surgical information, as well as the molecular data, whenever available [8,9,10]. 

Accordingly, DTCs with gross extra-thyroidal extension are considered at high risk of recurrence, intraglandular DTCs with non-aggressive histological features are considered as low-risk, while the remaining DTCs are categorized as intermediate-risk DTCs. The estimations of structural incomplete response ranges are between 10 and 55%, 1 and 5%, and between 3 and 30%, for high-, low-, and intermediate-risk DTC, respectively [6]. In clinical practice, the ATA systems proposed in 2009 [11] and amended in 2015 [6] appear to be an easy-to-use tool to assess patients’ risk of relapse [12]. However, these guidelines [6] contained some statements that, unfortunately, could not be backed up by high-quality evidence, and thus were based on low-quality data, as well as on the opinion of the experts’ board [13,14]. Moreover, these indications were not validated before their publication, and a significant overlap among the three risk categories exists [6].

Notably, the elements that were used to attain a risk stratification were based either on pre-operative staging, on macroscopic pathology findings, or, in some cases, on the molecular classification. The information provided by molecular imaging was never accounted for. This selection might be considered a shortcoming of the method, since radionuclide imaging can identify residual thyroid tissue, as well as microscopic metastatic spread, even when the morphological findings are normal. This information does not require performing additional diagnostic procedures but can simply be derived by observing the bio-distribution of radioiodine in patients undergoing adjuvant radioisotope ablation using an iodine isotope, namely ^131^I.

Recent data demonstrated that parameters derived from radioiodine therapy (RAI) and so-called peri-RAI findings (e.g., radioactive uptake outside the thyroid bed on post-therapeutic ^131^I-whole-body scan and thyroglobulin values measured before RAI), which are not included in the ATA risk classification, can grant a better stratification of the structural relapse risk in DTC patients [15]. This improvement was observed mainly in those patients who were deemed at low or intermediate risk after surgery, which were often correctly reclassified at a higher risk of relapse [16]. Thus, in the era of tailored medicine, with the introduction of the imaging-based scoring system, it would be required to identify a patient-adapted risk stratification to improve the individual patient’s management. This consideration caused us to develop a new multiparametric scoring system, including the ATA and the peri-RAI data. Other than the tumor-specific characteristics, further patient-specific features, such as age at diagnosis and gender, were demonstrated as independent predictors of disease recurrence and disease-specific mortality [17,18,19,20].

According to the above considerations, we hypothesized that pooling the single parameters into different domains could accurately estimate the risk of relapse of DTC patients. Since we considered that each domain included at least two risk factors describing one disease dimension, a three-domain model was designed, including the demographic, the surgical, and the RAI-related patients’ aspects. The present study was undertaken to (1) build a numerical scoring model to predict the risk of the recurrence of DTC accurately, and (2) to validate it in an extensive series of consecutive cases.

## 2. Materials and Methods

### 2.1. Study Design and Population

The historical dataset of DTC patients treated and followed up at the Nuclear Medicine Department of the E.O. Ospedali Galliera (Genoa, Italy) between 1992 and 2017 was considered. As previously reported, the reliability of the ATA risk stratification system was proven in this dataset thus assuring both the quality of the database and the validation of the ATA system (16). Briefly, patients were included when they were initially treated by total thyroidectomy (plus lymph-node neck dissection, whenever necessary) and RAI (range 2.96–7.4 GBq), which was administered after thyroid hormone withdrawal (THW). Conversely, patients with a follow-up time shorter than one year, with positive thyroid antibodies, or incomplete follow-up data were excluded. All selected DTC were reclassified according to the latest TNM version [17]. The institutional review board (Comitato Etico Regionale Liguria, Registration Number: 325/2020-DB id 10315) approved this retrospective study, and the requirement to obtain informed consent was waived. 

### 2.2. Definition of the Domains

The following domains were considered, based on the consensus among the authors and according to the available evidence-based literature: demographic, surgical, and RAI-related. 

The demographic domain included the patient’s age at diagnosis (e.g., > or <55 years) and gender. The surgical domain comprises the histological type (e.g., papillary, or follicular carcinoma) and the ATA risk category (low-risk, intermediate-risk, and high-risk) [1]. The RAI-related domain encompasses the serum thyroglobulin (Tg) value measured just before RAI (pre-RAI Tg) in THW condition (<5, 5–10, 10–50, >50 ng/mL) and findings on the post-therapeutic (PT) ^131^I WBS (e.g., remnant uptake only, neck uptake outside the thyroid bed, evidence of distant metastases). 

### 2.3. Study Reference Standards

Patients were classified as alive with no evidence of structural disease (NESD) when they were proven to have an “excellent response”, “biochemical incomplete response” or “indeterminate response”, according to the 2015 ATA guidelines [6]. Patients with a structural disease (e.g., cytology/histology and/or morphological/molecular imaging findings) accompanied by detectable Tg were classified as structural recurrence (REC). Progression-free survival (PFS) was calculated from the date of RAI to the date of the last follow-up visit in NESD patients, and from the date of RAI to the date of the detection of structural relapse in REC patients.

### 2.4. Statistical Considerations

The whole sample of 907 patients was randomly split into training and validation sets with an allocation ratio of 7:3. Statistical analyses were carried out on the training set and their predictive performance was assessed on the validation set using Harrell’s C-Index and its 95% confidence interval. Data were analyzed using common descriptive statistics as absolute numbers and frequencies, according to PFS. Fisher’s exact test was used to establish if each factor was eligible for the definition of the score.

The impact of each selected variable on PFS was assessed using a multivariate Cox model. The log-hazard ratio (HR) resulting from this analysis was then proportionally scaled to fit a 0–100 scale: the reference HR value of “1” was set to 0 points, and each HR increment beyond 1 imparted additional points, based on the HR value and on the weight of the factor. The total score of each patient was calculated as a sum of the three-domain sub-scores and ranged between 0 (lowest risk) and 100 (highest risk). This score was then stratified according to its terciles and the patients were sorted into three categories: mild risk 0–33, moderate risk 34–66, and severe risk 67–100. Statistical significance was set at 5%. All analyses were performed by STATA software (StataCorp. 2015. Release 14.2. College Station, TX: Stata Corp LP).

## 3. Results

### 3.1. Characteristics of the Series

According to the above selection criteria, 1146 DTCs were initially enrolled in the study. Among these, 239 patients were excluded due to a follow-up shorter than one year (*n* = 101), positive thyroid antibodies (*n* = 108), or incomplete data (*n* = 30). The final study series included 907 DTC patients. The characteristics of the subjects are summarized in Table 1. After the randomization, 635 patients were sorted into the training set (70%), while the remaining patients were assigned to the validation group (*n* = 272, 30%). The two sets were entirely comparable concerning the outcome and the prognostic factors (data not shown).

### 3.2. Training Set

The median follow-up was of 7.9 years. One hundred and forty (22.1%) patients showed a disease progression during the follow-up. Characteristics of the 635 subjects in the training set according to the PFS are listed in Table 2. Male gender, follicular histology, high ATA risk category, high pre-RAI Tg level, and the presence of metastases on PT ^131^I WBS were significantly related to an increased risk of disease progression (*p* < 0.01). Patients aged over 55 were more frequent in the group of events, even if this relation held up only weakly at the univariate level. Using the COX-PH model (Table 3), all the potential prognostic factors which resulted were significantly associated with the outcome. The hazard ratios of the prognostic factors were used to define the contribution of each of them to the three-domain score (Table 4). According to the ATA classification and the RAI-related domains, a high risk was the factor that resulted in the most relevant score calculation. According to the new score, 438 patients were classified as being at mild risk (69%), 125 at moderate risk (19.6%), and 72 at severe risk (11.4%). The obtained score was associated with the progression of the disease: patients with disease persistence or progression had, on average, 41.4 points more than those without (18.4 vs. 59.5 *p* < 0.001). For each unit increase in the score, the risk ramped up by 5.5% (95%CI 4.8–6.1, *p* < 0.001). 

### 3.3. Validation Set

**Figure 1:**
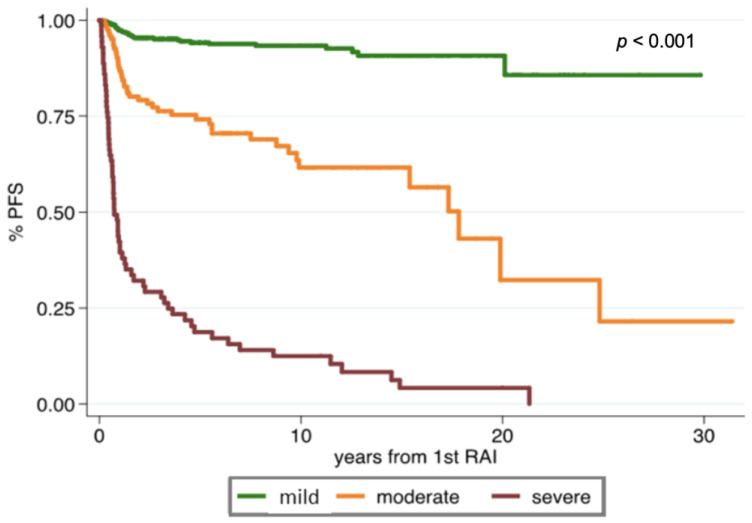
Kaplan–Meier curves of progression-free survival of the three-domain scoring system.

The division of the score in three groups identified three significantly different survival curves, as shown in Figure 1 (*p* < 0.001). Patients with moderate risk showed a likelihood of progression of about six times more than patients at mild risk (HR = 5.8, 95%CI = 3.6–9.3, *p* < 0.001) while the severe group presented a risk that was around 30-fold that of the mild group (HR = 29.7, 95%CI = 19.1–46.3, *p* < 0.001). The expected median PFS was 25.7 (95%CI = 23.4–28.1), 12.8 (95%CI = 8.5–17.1), and 0.7 (95%CI = 0.0–2.2) years for mild, moderate and severe risk groups, respectively (Table 4). The predictive performance of the new score was good, with a Harrell’s C-index of 83.1 (95%CI = 77.7–88.5).

## 4. Discussion

In this study, we have identified and validated a large series of patients affected by DTC, and created a three-domain scoring system able to identify the risk of the persistence/relapse of disease after the standard upfront treatment, consisting of total thyroidectomy and RAI. This score is easy to calculate and interpret, as it provides a score ranging from 0 to 100; it allows clinicians to identify those patients who need more restricted clinical surveillance or an adaptation of the treatment protocol, due to a risk of disease persistence/relapse. This prognostic system can be used to semi-quantify the recurrence risk. This three-domain system includes two potential prognostic factors such as demographic (age and gender) and RAI-related (pre-RAI Tg levels and the post-therapeutic 131I WBS) data. The first is a recognized prognostic factor; in particular, there is ample evidence that younger patients have a much better prognosis than older patients [21,22]. As per the pre-RAI Tg levels and post-therapy imaging, they represent two different methods to estimate the disease burden. It is worth mentioning that the combined evaluation of these two parameters may also deliver valuable information regarding the disease aggressiveness, as is the case regarding the discrepancy between the tumor marker levels and the evidence of iodine-active tissue [23,24].

The ATA risk stratification system does mention the peri-RAI parameters as a tool to tailor the therapeutic management of DTC. However, in the ATA guidelines, the recommendation 51 (summarized in the Table 14, located at the page 56 of the document) states that the indication for RAI should only consider TNM staging and peri-operative features [6]. In addition, only a vague hint to the high levels of pre-RAI-Tg has been included as a possible method to identify the high-risk patients, by mentioning subjects with “preoperative serum Tg suggestive for metastases.” Indeed, a reliable pre-RAI-Tg threshold, able to guide the clinicians in recognizing DTC patients at higher risk, is not entailed within the definition of Tg “suggestive for metastases.” In this proposed three-domain system, we have considered the prognostic role of pre-RAI-Tg accurately by introducing, according to the literature [16,25,26,27,28,29], well-defined cutoff values of Tg >10 and >50 ng/mL, identifying patients with a higher likelihood of extrathyroidal or distant disease foci, who therefore present an increased risk of DTC persistence or relapse.

At the same time, we have considered an easy-to-implement classification of the PT-^131^I-WBS (e.g., remnant uptake, uptake outside thyroid bed, and iodine avid distant metastases) that correctly stage the patients, providing essential information on the risk of disease persistence/relapse [30,31], as well as on the degree of differentiation of said localizations.

These last prognostic parameters are, of course, only available in the case of RAI therapy followed by PT-^131^I-WBS or in the case of a diagnostic pre-therapeutic ^131^I/^123^I WBS with SPECT/CT [32,33,34]. They have recently demonstrated reliability in the stratification of the DTC relapse risk, especially in the context of ATA low-risk or intermediate-risk patients [16]. These recent data showed that about 30% of patients at low-intermediate risk on ATA classification showed a higher risk of disease relapse when the RAI-related parameters were factored in. Interestingly, the risk of the structural incomplete response of ATA low-risk patients was 7%, while the frequency observed in patients classified according to the RAI-related findings was 13%. Likewise, when the intermediate-risk ATA category was reclassified in the intermediate risk+ category, according to the peri-RAI information, it was observed that the latter subcategory entailed a significantly higher recurrence rate of 36.6%, almost three times higher than the intermediate class without additional risk factors (13.3%) [16].

In the present paper, owing to the three-domain system, we identified patients with significantly different classes of disease relapse risk (Figure 1), and we confirmed the pivotal prognostic role of peri RAI findings, which, as per Table 4, had a greater impact on the final score when compared with the other parameters. This aspect seemed to be of importance, in particular, in low- to intermediate-risk DTC patients according to the TNM classification [17]. 

Although our results are encouraging, we should bear in mind some limitations. Firstly, this was a retrospective study, which included only patients treated with RAI. A control population of patients not treated with RAI could have likely better defined the reliability of our scoring system. Such an evaluation would be of interest, considering the increasing tendency of treating DTC with lobectomy alone, observed in recent years. Secondly, the patients were all treated and followed up in a single institution. Validation on a different population treated in a different center would have been useful to confirm the impact of this scoring system. However, the monocentric nature of this study allowed the inclusion of a homogenously treated population. Thirdly, given the very long study period, the patients included could have been managed according to slightly different strategies and the Tg assay methods could have been changed over time. Considering a long period of 25 years, only the information of planar iodine ^131^I-WBS was included. However, we have only classified as positive those patients with clear evidence of radioactive uptake outside the thyroid bed, and, therefore, we have probably underestimated the involvement of small lymph nodes. Within the ever-changing landscape of DTC, there are many other patient-, disease, and treatment-related factors, such as TSH modulation, the presence of concomitant autoimmune disease, and molecular factors, which can play a relevant role in the outcome prediction [35,36,37]. However, in constructing the proposed score, we focused on those factors which had the most extensive validation and reproducibility; the scoring system was made as easy to implement as possible. The proposed system was apt in the evaluation of disease persistence and recurrence-free survival, though it was not automatically translatable to the context of overall survival.

Finally, this system might not have been fully applicable to patients with evidence of anti-Tg antibodies because of the low reliability of the Tg measurements in this scenario.

## 5. Conclusions

Managing DTC patients, according to their demographic, surgical, and radioiodine-related domains, can help to stratify their cancer relapse risk. We recommend conducting further studies, with perspective design, on this topic.

## Figures and Tables

**Table 1:** Characteristics of the study cohort.

Variable	Subjects Included (*n* = 907)
Sex	
Female, no. (%)	643 (70.9)
Male, no. (%)	264 (29.1)
Age on diagnosis, median (IQR), years	53 (41–63)
<55 years, no. (%)	485 (53.5)
≥55 years, no. (%)	422 (46.5)
Pathology	
Papillary thyroid carcinoma, no. (%)	791 (87.2)
Follicular thyroid carcinoma, no. (%)	116 (12.8)
Clinico-pathological classification *	
T1, no. (%)	458 (50.5)
T2, no. (%)	137 (15.1)
T3, no. (%)	247 (27.2)
T4, no. (%)	65 (7.2)
N0, no. (%)	669 (73.8)
N1, no. (%)	238 (26.2)
M0, no. (%)	887 (97.8)
M1, no. (%)	20 (2.2)
TSH after THW, media (IQR), uIU/ml	65.5 (49.2–89.7)
Radioactive iodine dose, median (IQR), GBq	2.96 (2.96–3.7)
Outcome	
No evidence of structural disease, no. (%)	674 (74.3)
Recurrence, no. (%)	193 (21.3)
Death, no. (%)	40 (4.4)

* This feature included all histopathological findings and pre-surgical imaging.

**Table 2:** Association between each parameter of the three domains and progression-free survival.

Descriptive Statistics (Absolute Number and Frequencies) According to the Onset of PFS during the Follow-up in the Training Set.			PFS			*p*-Value *
			Overall	No	Yes	
			635 (100%)	495 (78.0)	140 (22.1)	
Demographic domains	Age at diagnosis	<55	353 (55.6)	283 (57.2)	70 (50.0)	0.149
		≥55	212 (42.8)	212 (42.8)	70 (50.0)	
	Sex	F	441 (69.5)	362 (73.1)	79 (56.4)	< 0.001
		M	194 (30.5)	133 (26.9)	61 (43.6)	
Surgical domains	Histological type	Papillary CA	549 (86.5)	441 (89.1)	108 (77.1)	0.001
		Follicular CA	86 (13.5)	54 (10.9)	32 (22.9)	
	ATA risk category	Low	341 (53.7)	321 (64.8)	20 (14.3)	<0.001
		Intermediate	217 (34.2)	148 (29.9)	69 (49.3)	
		High	77 (12.1)	26 (5.2)	51 (36.4)	
RAI-related domains	Pre-RAI-Tg (ng/mL)	(0–5)	392 (61.7)	364 (73.5)	28 (20.0)	<0.001
		(5–10)	75 (11.8)	63 (12.7)	12 (8.6)	
		(10–50)	90 (14.2)	59 (11.9)	31 (22.1)	
		>50	78 (12.3)	9 (1.8)	69 (49.3)	
	Post-therapeutic ^131^I WBS	R	549 (86.5)	473 (95.6)	76 (54.3)	<0.001
		Outside thyroid bed	55 (8.7)	21 (4.2)	34 (24.3)	
		Distant metastases	31 (4.9)	1 (0.2)	30 (21.4)	

* *p*-values referred to Fisher’s Exact test for frequencies.

**Table 3:** The Cox model was calculated to confirm the significant effect of each prognostic parameter on the outcome.

Prognostic Parameters	Model Estimates	
	Hazard Ratio * (95%CI)	*p*-Value
Progression-Free Survival		
Histology		
Papillary	1 (reference)	
Follicular	1.57 (1.0–2.57)	0.047
2015 ATA risk category		
Low-Risk	1 (reference)	
Intermediate-Risk	4.25 (2.52–7.14)	<0.001
High-Risk	4.52 (2.45–8.33)	<0.001
Tg measured before RAI in THW (ng/mL)		
(0–5)	1 (reference)	
(5–10)	1.47 (0.72–2.97)	0.28
(10–50)	3.58 (2.11–6.05)	<0.001
>50	7.15 (4.11–12.44)	<0.001
Post-therapeutic WBS findings		
Only remnant uptake	1 (reference)	
Uptake outside thyroid bed	2.49 (1.56–3.96)	<0.001
Distant metastases	3.23 (1.76–5.92)	<0.001

* Adjusted for age and sex.

**Table 4:** Calculation of the three-domain scoring system.

Domains	Risk Factors	Levels				Score
Demographic	Age	<55		≥55		
		0		4		
	Sex	Female		Male		
		0		8		
Surgical	Histology	Papillary Thyroid cancer		Follicular Thyroid cancer		
		0		8		
	ATA 2015	Low	Intermediate	High		
		0	25	26		
RAI related	Tg	(0–5)	(5–10)	(10–50)	≥50	
		0	7	22	34	
	PT-WBS	Only remnant uptake	Uptake outside the thyroid bed	Distant metastases		
		0	16	20		
				TOTAL		
	Risk categories		Mild risk	Moderate risk	Severe risk	
			0–33	34–66	67–100	
	Median PFS years (95%CI)		25.7 (23.4–28.1)	12.8 (8.5–17.1)	0.7 (0.0–2.2)	

## Data Availability

Data regarding the present study are available at the authors’ institution and can be obtained upon reasonable request.

## Abbreviations

ATA	American Thyroid Association
DTC	Differentiated Thyroid Cancer
HR	Hazard ratio
NESD	No evidence of structural disease
PFS	Progression-free survival
PT	Post-therapy
RAI	Radioactive Iodine
REC	Structural recurrence
SPECT/CT	Single-photon emission computed tomography/X-ray computed tomography
Tg	Thyroglobulin
THW	Thyroid Hormone Withdrawal
TNM	Tumour-node-metastases
WBS	Whole-body scan
